# Supplementary figures and images for: Complete genome sequencing and characterization of a potential new genotype of Citrus tristeza virus in Iran
Source: PLoS One. 2023 Jun 29;18(6):e0288068. doi: 10.1371/journal.pone.0288068 (PMC10310044; doi:10.1371/journal.pone.0288068)

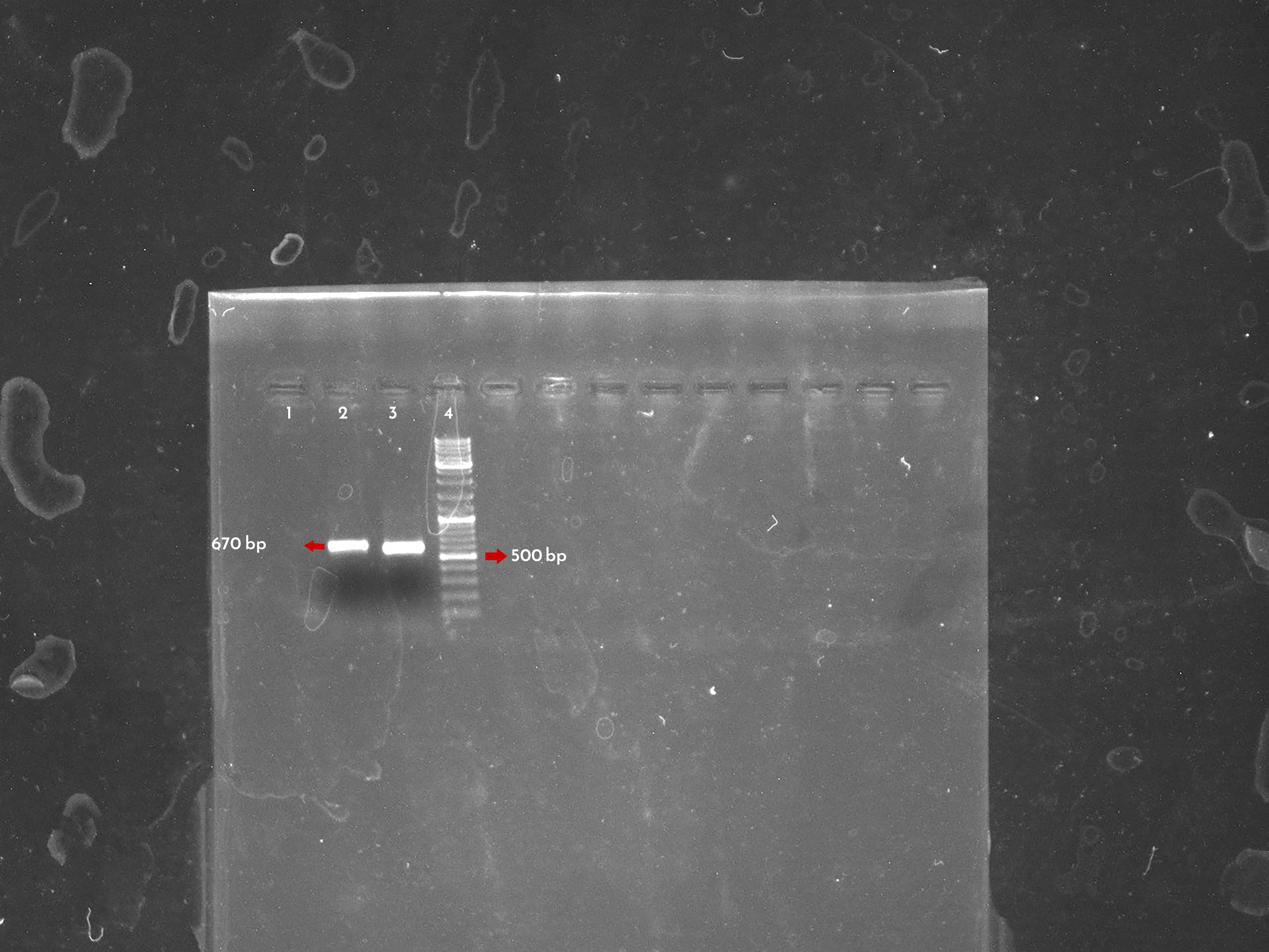

Supplement: S1 Raw images — (TIF) [file pone.0288068.s002.tif]
